# Reinforcement of transcriptional silencing by a positive feedback between DNA methylation and non-coding transcription

**DOI:** 10.1093/nar/gkab746

**Published:** 2021-09-01

**Authors:** M Hafiz Rothi, Masayuki Tsuzuki, Shriya Sethuraman, Andrzej T Wierzbicki

**Affiliations:** Department of Molecular, Cellular, and Developmental Biology, University of Michigan, Ann Arbor, MI 48109, USA; Department of Molecular, Cellular, and Developmental Biology, University of Michigan, Ann Arbor, MI 48109, USA; Department of Life Sciences, Graduate School of Arts and Sciences, The University of Tokyo, Meguro-ku, Tokyo 153-8902, Japan; Bioinformatics Graduate Program, University of Michigan, Ann Arbor, MI 48109, USA; Department of Molecular, Cellular, and Developmental Biology, University of Michigan, Ann Arbor, MI 48109, USA

## Abstract

Non-coding transcription is an important determinant of heterochromatin formation. In *Arabidopsis thaliana* a specialized RNA polymerase V (Pol V) transcribes pervasively and produces long non-coding RNAs. These transcripts work with small interfering RNA to facilitate locus-specific establishment of RNA-directed DNA methylation (RdDM). Subsequent maintenance of RdDM is associated with elevated levels of Pol V transcription. However, the impact of DNA methylation on Pol V transcription remained unresolved. We found that DNA methylation strongly enhances Pol V transcription. The level of Pol V transcription is reduced in mutants defective in RdDM components working downstream of Pol V, indicating that RdDM is maintained by a mutual reinforcement of DNA methylation and Pol V transcription. Pol V transcription is affected only on loci that lose DNA methylation in all sequence contexts in a particular mutant, including mutants lacking maintenance DNA methyltransferases, which suggests that RdDM works in a complex crosstalk with other silencing pathways.

## INTRODUCTION

RNA-directed DNA methylation (RdDM) is a plant transcriptional silencing pathway which targets transposable elements (TE), transgenes and repetitive sequences (1). These loci are then turned off by the establishment of repressive chromatin marks, including posttranslational histone modifications, nucleosome positioning and DNA methylation (2,3). RdDM is determined by two classes of non-coding RNA (4–6). The first is small interfering RNA (siRNA), which is produced by the activities of RNA-dependent RNA polymerases and Dicer-like proteins (7). siRNA incorporates into Argonaute proteins and gives them sequence-specificity towards loci complementary to siRNA (8,9). The second class of non-coding RNA involved in RdDM is produced by a specialized RNA polymerase, Pol V (10–14). Pol V transcribes long non-coding RNA (lncRNA) and lncRNA is required for recognition of target loci by siRNA-Argonaute complexes, which has been proposed to occur via siRNA-lncRNA base-pairing (9,11,15,16). The consequence of this recognition is recruitment of chromatin modifiers and heterochromatin formation (17–19).

The most important property of RdDM is its locus specificity, which assures that TEs are recognized and silenced, but essential protein-coding genes are not targeted. This specificity is achieved when a TE is newly integrated or activated. As a TE becomes transcribed by Pol II, it produces aberrant transcripts, which are the preferred substrates for RNA-dependent RNA polymerases and give rise to primary siRNAs (7,20). Pol V has been recently shown to transcribe broadly and surveil the genome to make it competent to receive the silencing signal from primary siRNA (12). Therefore, Pol V contributes little or no sequence-specificity to the initiation of RdDM.

Once initiated, silencing is often not maintained epigenetically and has to be reinforced by a continuous activity of the RdDM pathway. This process involves another specialized RNA polymerase, Pol IV, which produces substrates for RDR2 and DCL3 and leads to relatively high accumulation of 24nt siRNA (21,22). It also involves Pol V, which transitions from a very low level of surveillance transcription to a more efficient production of lncRNAs on silenced loci (12). Both events are caused by the presence of repressive chromatin marks. H3K9me2 is recognized by the SHH1 protein, which recruits Pol IV (23,24). Methylated DNA is bound by SUVH2 and SUVH9 proteins, which facilitate Pol V transcription (25,26). Consistently, Pol V association with chromatin is often reduced in the *met1* mutant (25). This strongly suggests that RdDM is a self-reinforcing mechanism, where DNA methylation and H3K9me2 enhance Pol IV and Pol V transcription, which leads to further reestablishment of repressive chromatin marks.

The presence of a self-reinforcing feedback loop between elevated Pol V transcription and DNA methylation has one important implication. It suggests that disruption of RdDM factors that work downstream of Pol V should lead to loss of DNA methylation and subsequently reduction of Pol V transcription. Surprisingly, it is not the case and Pol V transcripts still accumulate in those mutants, including *spt5l*, *ago4* and *drm2* (9,11–13,17,27). This inconsistency indicates that the relationship between Pol V transcription and DNA methylation remains unresolved.

One possible explanation for the inability to disrupt the RdDM feedback loop is the presence of multiple overlapping silencing pathways (25,28–31). In this scenario, maintenance of silencing on a subset of RdDM loci may be performed not only by RdDM but also by MET1 and/or CMT3. In this study we tested this possibility by performing genome-wide identification of Pol V transcription in mutants defective in downstream RdDM components and DNA methyltransferases. We found that loci transcribed by Pol V are indeed targeted by multiple overlapping and partially redundant silencing pathways. This confounds the ability to detect the self-reinforcing properties of RdDM. When effects of other pathways are eliminated, the positive feedback of Pol V transcription and DNA methylation becomes clearly detectable.

## MATERIALS AND METHODS

### Reagents

The antibody against the largest subunit of Pol V (NRPE1) was described previously (10,12,32).

### Biological resources

We used the following genotypes of *Arabidopsis thaliana*: Columbia-0 ecotype (wildtype), *nrpe1* (*nrpd1b-11* (33)), *ago4-1* (introgressed into the Col-0 background (9)), *spt5l* (SALK_001254), *drm2-2* (SAIL_70_E12), *cmt3-11* (SALK_148381), and *met1-3* (34). Plants were grown at 22ºC under white LED light in 16 h/8 h day/night cycle.

### Computational resources

During data analysis we used bowtie2 2.2.9 (12), BEDTools 2.15.0 (35), the NBPseq R package (36), GFOLD (37), Bismark (38) and methylKit R package (39). *Arabidopsis* genome annotations (TAIR10) were obtained from TAIR (www.arabidopsis.org). Previously published high throughput sequencing datasets were obtained from Gene Expression Omnibus (https://www.ncbi.nlm.nih.gov/geo/). Pol V IPARE data (GSE146913) and annotated regions were published previously (12). TE regions annotated by RdDM categories were provided by the Slotkin lab (40,41). DNA methylation data (GSE39901) were obtained from previously published datasets (42).

### Statistical analyses

Significant differences in the levels of Pol V transcription were identified using Robinson and Smyth's exact negative binomial test implemented in the NBPseq R package (36) using data from two independent biological replicates. For the *met1* mutant significant differences in the levels of Pol V transcription were identified using generalized fold change algorithm implemented in GFOLD (37). Levels of DNA methylation or Pol V transcription on groups of genomic bins were compared using the Wilcoxon test.

### Pol V IPARE

Three grams of aerial tissue of 18-day old plants were used for Pol V IPARE experiments carried out as described (12). High throughput sequencing was performed at the University of Michigan Advanced Genomics Core.

### Bioinformatic analysis

Pol V IPARE sequencing reads were processed and aligned to the *Arabidopsis* TAIR10 genome with bowtie2 as described previously (12). Pol V IPARE levels were plotted as boxplots by counting the number of reads in studied genomic regions using BEDTools and normalized as number of reads per million mapped reads (RPM) (35). Information about IPARE datasets generated and used in this study is presented in Supplementary Table S1.

To identify regions differentially transcribed by Pol V, we counted the number of IPARE reads in 100 bp bins with a step-size of 50 bp across the whole genome. We then tested for differential Pol V transcription in the bins between Col-0 and specific mutants with false discovery rate (FDR) < 0.04 using NBPseq (36). Overlap analyses between Pol V IPARE reduced in *drm2* regions and specific genomic regions (Figure 6A) were performed with 1000 permutated genomic regions using BEDTools to obtain expected numbers and p-values (35). TE ends were defined as 150 bp at the end of TEs and TE inner are the remainders of annotated TEs. Average profiles of Pol V IPARE signal at ends of Pol V RdDM TEs with lengths of > 500 bp, were plotted with Col-0 divided by *drm2*. Reductions of Pol V transcription in *drm2*, *spt5l*, *ago4* and *cmt3* mutants was determined by FDR < 0.05. Pol V transcription was determined to be unchanged if FDR was greater than 0.9 and fold change smaller than 2. Reduction of Pol V transcription in *met1*, which was based on one replicate of Pol V IPARE was determined using GFOLD (37) with the *P* < 0.01 at 2-fold change or greater. Pol V transcription was determined to be unchanged in *met1* if *P* < 0.01 at 0.1-fold change or smaller and fold change smaller than 2.

Sequencing reads from whole genome bisulfite-seq datasets were mapped to the *Arabidopsis* TAIR10 genome using Bismark allowing no mismatches (38). DNA methylation levels were calculated by the ratio of #C/(#C+#T) after selecting for Cs with at least five sequenced reads. Differentially Methylated Regions (DMRs) were identified using methylKit (39). The bin sizes used were 100 bp bins with a step-size of 50 bp. A minimum of 5 reads was required for each cytosine. For *drm1/2* DMRs, 25% minimum difference in CHH context DNA methylation was selected for in each of the tiles with FDR < 0.01. For *met1* DMRs, 55% minimum difference in CG context DNA methylation was selected for in each of the tiles with FDR < 0.01. DNA methylation levels used as the cutoff for presence of each context in the DNA methylation categories in Figures 2–5 were 5% CHH, 10% CHG and 20% CG. DNA methylation levels used as the cutoff for absence of each context in the DNA methylation categories in Figures 2–5 were 0% CHH, 0% CHG and 0% CG in the respective mutant that was tested.

## RESULTS

### RdDM loci are targeted by multiple silencing pathways

**Figure 1.**
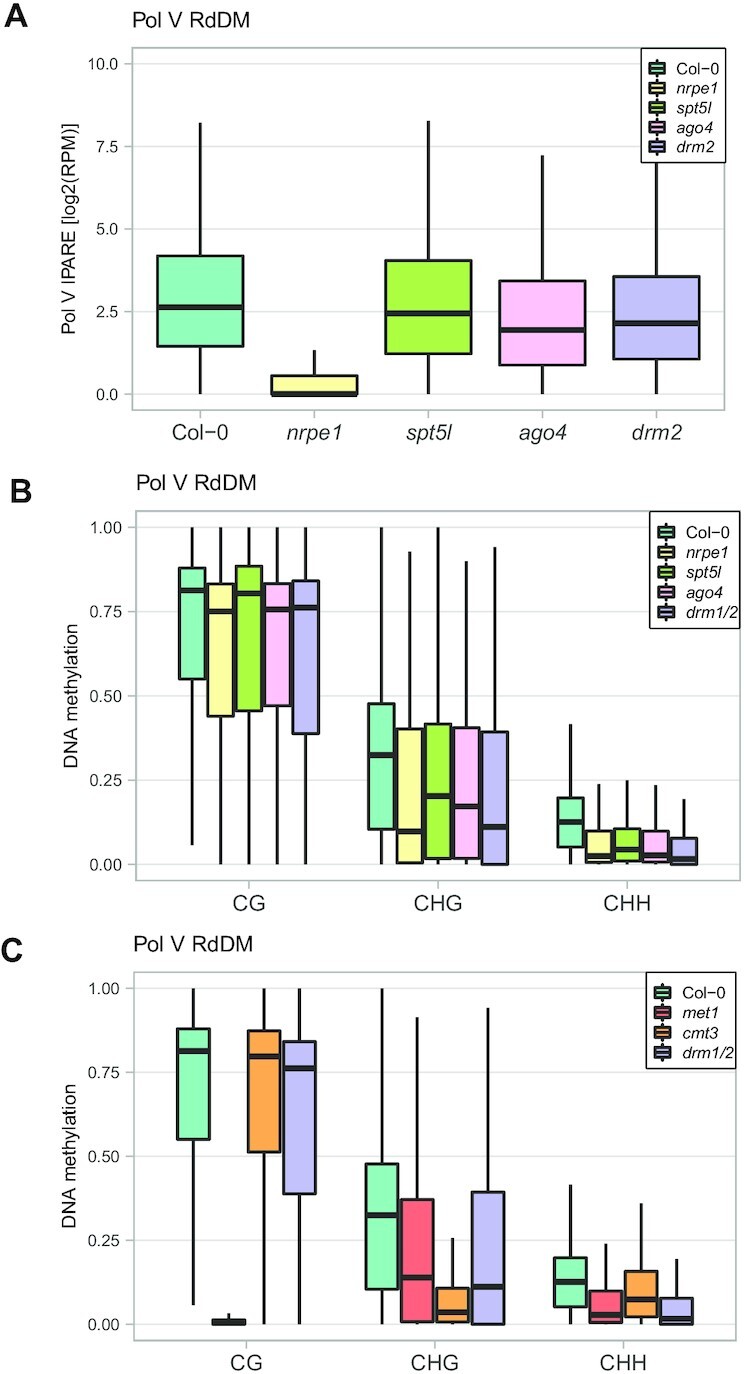
RdDM loci are targeted by multiple silencing pathways. (**A**) Small effects of mutants in downstream components of RdDM on Pol V transcription throughout the genome. Pol V IPARE signal levels were plotted on previously identified Pol V RdDM regions (12) in Col-0, *nrpe1*, *spt5l*, *ago4*, and *drm2*. Individual biological replicates are shown in Supplementary Figure S1AB. (**B**) Presence of symmetric DNA methylation in RdDM mutants. DNA methylation levels (42) were plotted on previously identified Pol V RdDM regions (12) in CG, CHG and CHH contexts in Col-0, *nrpe1*, *spt5l*, *ago4*, and *drm1/2*. (**C**) Residual DNA methylation in DNA methyltransferase mutants. DNA methylation levels (42) were plotted on previously identified Pol V RdDM regions (12) in CG, CHG and CHH contexts in Col-0, *met1*, *cmt3* and *drm1/2*.

RdDM has been proposed to work as a self-reinforcing feedback loop (25), which predicts that mutants in components acting downstream of Pol V should affect the accumulation of Pol V transcripts. Several studies indicated that this is not the case and Pol V transcripts accumulate in *spt5l*, *ago4* and *drm2* mutants (9,11–13,17,27). To resolve these conflicting results, we performed Pol V IPARE in the *drm2* mutant, and reanalyzed previously published comparable Pol V IPARE datasets in Col-0, *ago4* and *spt5l* (12). As expected, the overall accumulation of Pol V transcripts on all known RdDM Pol V-transcribed regions (12) was only slightly reduced in *spt5l*, *ago4* or *drm2* mutants. This reduction was much smaller than observed in *nrpe1*, a mutant in the largest subunit of Pol V (Figure 1A, Supplementary Figure S1AB).

One potential explanation of this observation is that not all DNA methylation is lost in the studied mutants (29). To test this hypothesis, we reanalyzed previously published whole genome bisulfite sequencing datasets (42) and determined the levels of DNA methylation in all three contexts on the same known RdDM Pol V-transcribed regions (12). We found that while CHH methylation was substantially reduced, the levels of CG methylation remained high in *spt5l*, *ago4* and *drm1/2* mutants (Figure 1B). The remaining CG methylation may explain why these mutants only have minor effects on Pol V transcription.

High levels of residual DNA methylation in RdDM mutants are consistent with previous observations that RdDM loci are commonly targeted by several silencing pathways (25,30,31). To provide further support for this conclusion, we determined the levels of DNA methylation on RdDM Pol V-transcribed regions (12) in DNA methyltransferase mutants, which disrupt various silencing pathways. The *cmt3* mutant had a strong reduction of CHG methylation only (Figure 1C). *drm1/2* had reduced levels of CHH and to a smaller extent CHG methylation but no major change in CG methylation (Figure 1C). *met1* had an almost complete loss of CG methylation but only partial reductions of CHH and CHG methylation (Figure 1C). This indicates that as expected, RdDM Pol V-transcribed loci are targeted not only by RdDM but also by variable contributions of CMT3 and MET1. Together, these results indicate that RdDM loci are targeted by multiple overlapping silencing pathways.

### Maintenance of RdDM requires DNA methylation by DRM2

**Figure 2.**
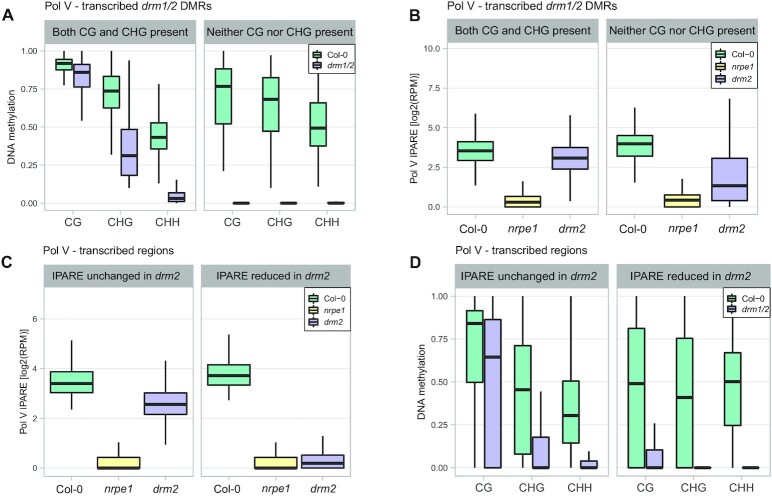
Maintenance of RdDM requires DNA methylation by DRM2 at loci not targeted by other silencing pathways. (**A**) Control plot showing *drm1/2* DMRs split by the presence or absence of symmetric methylation in *drm1/2*. DNA methylation levels (42) were plotted on Pol V-transcribed *drm1/2* CHH DMRs split by the levels of CG and CHG methylation. There were 2035 DMRs with both CG and CHG present in both Col-0 and *drm1/2* as well as 176 DMRs with CG and CHG present in Col-0 but absent in *drm1/2*. DMRs were identified by difference between the whole genome bisulfite sequencing (WGBS) CHH signal of Col-0 and *drm1/2* > 0.2 and FDR < 0.01. Presence of DNA methylation was defined as WGBS signal > 0.2 (CG) or > 0.1 (CHG). Absence of DNA methylation was defined as WGBS signal of 0. (**B**) Substantial reduction of Pol V transcription in *drm2* on loci that lose DNA methylation in all contexts. Pol V IPARE signal was plotted on two categories of Pol V-transcribed *drm1/2* DMRs in Col-0, *nrpe1*, and *drm2*. Individual biological replicates are shown in Supplementary Figure S2AB. (**C**) Control plot showing genomic Pol V-transcribed bins split by the impact of DRM2 on Pol V transcription. Pol V IPARE signal was plotted on Pol V-transcribed regions with either Pol V IPARE reduced (1246 bins) or unchanged (8945 bins) in *drm2*. Bins were identified as Pol V-transcribed by IPARE signal being significantly greater in Col-0 compared to *nrpe1* (FDR < 0.05 (36)). IPARE signal was defined as reduced in *drm2* by FDR < 0.05, and as unchanged in *drm2* by FDR > 0.9 and fold change smaller than 2. Individual biological replicates are shown in Supplementary Figure S2C. (**D**) Substantial reduction of DNA methylation in *drm1/2* in all contexts on genomic bins with DRM2-dependent Pol V transcription. DNA methylation levels (42) in CG, CHG and CHH contexts were plotted on Pol V-transcribed regions with Pol V IPARE signal reduced or unchanged in *drm2*. Corresponding data for *nrpe1* and total levels of DNA methylation in all contexts are shown in Supplementary Figure S2D.

Presence of multiple silencing pathways on RdDM loci may confound the ability to test the role of DNA methylation for Pol V transcription. To overcome this limitation, we took advantage of the fact that each particular locus may be targeted by any combination of silencing pathways and relative contributions of various pathways at least partially depend on the frequency of cytosines in particular contexts (31). This means that some loci may be primarily silenced by just one pathway and therefore a subset of loci is expected to have no DNA methylation in *drm2* in all contexts. To identify these loci, we found differentially methylated regions (DMRs) that lose CHH methylation in *drm1/2* (*drm1/2* DMRs) and are transcribed by Pol V. We then split these DMRs into two categories based on the presence or absence of CG and CHG methylation in *drm1/2*. The control group had CG and CHG methylation detectable in *drm1/2* (Figure 2A, ‘Both CG and CHG present’). The second group had no CHG and no CG methylation detectable in *drm1/2* (Figure 2A, ‘Neither CG nor CHG present’). We then calculated the abundance of Pol V transcription in those groups in Col-0 wild type and *drm2* mutant. While the control group had only a small reduction of Pol V transcription in *drm2* (Figure 2B), the group with no CHG and no CG methylation had a substantially greater reduction of Pol V transcription in *drm2* (Figure 2B, Supplementary Figure S2AB). The level of Pol V transcription in *drm2* on loci with no CHG and no CG methylation in *drm1/2* was significantly lower than on control loci (*P* < 10^–16^, Wilcoxon test). This indicates that loss of DNA methylation in all contexts in *drm2* leads to a substantial reduction of Pol V transcription.

To further confirm the role of all DNA methylation contexts for maintaining high levels of Pol V transcription, we performed a reciprocal analysis. We identified Pol V-transcribed genomic regions, where Pol V IPARE signal was significantly reduced in *drm2* and control loci where no difference in Pol V IPARE signal was detected in *drm2* (Figure 2C, Supplementary Figure S2C). We then assayed DNA methylation in Col-0 wild type, *drm1/2* and *nrpe1*. Loci where Pol V transcription was DRM2-independent showed strong reductions of CHG and CHH methylation but mostly maintained relatively high levels of CG DNA methylation in *drm1/2* (Figure 2D). In contrast, loci that lost Pol V transcription in *drm2* also lost DNA methylation in all sequence contexts, including CG (Figure 2D). Levels of CG methylation in *drm1/2* on loci that lost Pol V transcription in *drm2* were significantly lower than at loci where Pol V transcription was DRM2-independent (*P* < 10^–179^, Wilcoxon test). This indicates that residual CG methylation allows maintaining high levels of Pol V transcription and the reduction of Pol V transcription in *drm2* is associated with the loss of DNA methylation in all sequence contexts. Levels of DNA methylation in all contexts were similar in *drm1/2* and *nrpe1* on both categories of loci (Supplementary Figure S2D), which is consistent with Pol V being generally required for DNA methylation by DRM2.

Together, these results indicate that RdDM Pol V transcription requires DNA methylation in at least one sequence context. This is consistent with RdDM operating as a self-reinforcing feedback loop and enhanced Pol V transcription on silenced loci playing an important role in this feedback.

### Downstream components are required for maintenance of RdDM

**Figure 3.**
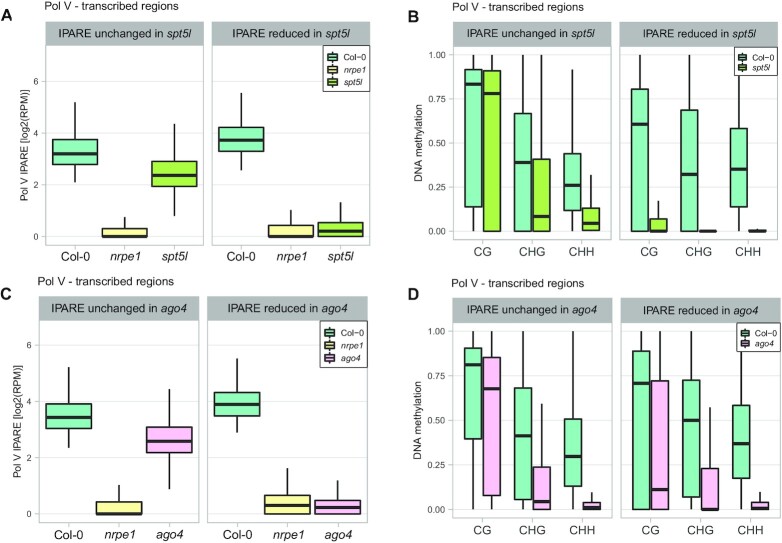
Downstream components are required for maintenance of RdDM at loci where they are needed for DNA methylation in all contexts. (**A**) Control plot showing Pol V-transcribed genomic bins split by the impact of SPT5L on Pol V transcription. Pol V IPARE signal was plotted on Pol V transcribed regions with either Pol V IPARE reduced (1304 bins) or unchanged (13115 bins) in *spt5l*. Bins were identified as Pol V-transcribed by IPARE signal being significantly greater in Col-0 compared to *nrpe1* (FDR < 0.05 (36)). IPARE signal was defined as reduced in *spt5l* by FDR < 0.05, and as unchanged in *spt5l* by FDR > 0.9 and fold change smaller than 2. Individual biological replicates are shown in Supplementary Figure S3A. (**B**) Substantial reduction of DNA methylation in *spt5l* in all contexts on genomic bins with SPT5L-dependent Pol V transcription. DNA methylation levels (42) in CG, CHG and CHH contexts was plotted on regions with Pol V IPARE signal reduced or unchanged in *spt5l*. (**C**) Control plot showing genomic bins split by the presence or absence of AGO4-dependent Pol V transcription. Pol V IPARE signal was plotted on regions with either Pol V IPARE reduced (1048 bins) or unchanged (9181 bins) in *ago4*. Bins were identified as Pol V-transcribed by IPARE signal being significantly greater in Col-0 compared to *nrpe1* (FDR < 0.05 (36)). IPARE signal was defined as reduced in *ago4* by FDR < 0.05, and as unchanged in *ago4* by FDR > 0.9 and fold change smaller than 2. Individual biological replicates are shown in Supplementary Figure S3B. (**D**) Substantial reduction of DNA methylation in *ago4* in all contexts on genomic bins with AGO4-dependent Pol V transcription. DNA methylation levels (42) in CG, CHG and CHH contexts were plotted on Pol V-transcribed regions with Pol V IPARE signal reduced or unchanged in *ago4*.

The self-reinforcing loop between Pol V transcription and DNA methylation is expected to be disrupted not only in the *drm2* mutant but also in mutants defective in other downstream RdDM components, including *spt5l* and *ago4*. To test this prediction, we analyzed Pol V IPARE from the *spt5l* mutant (12) and identified Pol V-transcribed genomic regions that had no changes of Pol V transcription in *spt5l* (Figure 3A, Supplementary Figure S3A). These regions had strong reductions of CHG and CHH methylation but retained high levels of CG methylation in *spt5l* (Figure 3B). In contrast, regions with significant reductions of Pol V transcription in *spt5l* (Figure 3A, Supplementary Figure S3A) had substantial reductions of DNA methylation in all sequence contexts, including CG (Figure 3B). Levels of CG methylation in *spt5l* at loci that lost Pol V transcription in *spt5l* were significantly lower than at loci where Pol V transcription was SPT5L-independent (*P* < 10^–250^, Wilcoxon test). This indicates that residual CG methylation allows maintaining high levels of Pol V transcription and a subset of loci where the level of Pol V transcription is dependent on SPT5L also loses DNA methylation in all sequence contexts in *spt5l*.

We further tested the contribution of AGO4 to the self-reinforcement of RdDM by analyzing Pol V IPARE in the *ago4* mutant. Pol V-transcribed genomic regions with no reductions of Pol V transcription in *ago4* (Figure 3C) had strong reductions of CHG and CHH methylation but retained high levels of CG methylation in *ago4* (Figure 3D). Regions that lost Pol V transcription in *ago4* (Figure 3C, Supplementary Figure S3B) also showed substantial reduction of DNA methylation in *ago4* in all sequence contexts, including CG (Figure 3D). Levels of CG methylation in *ago4* at loci that lost Pol V transcription in *ago4* were significantly lower than at loci where Pol V transcription was AGO4-independent (*P* < 10^–51^, Wilcoxon test). This further demonstrates the role of residual CG methylation in maintaining Pol V transcription and shows that a subset of loci where Pol V transcription is dependent on AGO4 also loses DNA methylation in *ago4* in all sequence contexts.

Together, these results demonstrate that Pol V transcription is enhanced by DNA methylation and confirm that RdDM is controlled by a self-reinforcing feedback loop between the level of Pol V transcription and DNA methylation. This feedback loop may be disrupted by mutating *SPT5L* or *AGO4* and is only detectable on loci with no confounding activity of other silencing pathways.

### MET1 is needed for maintenance of RdDM

**Figure 4.**
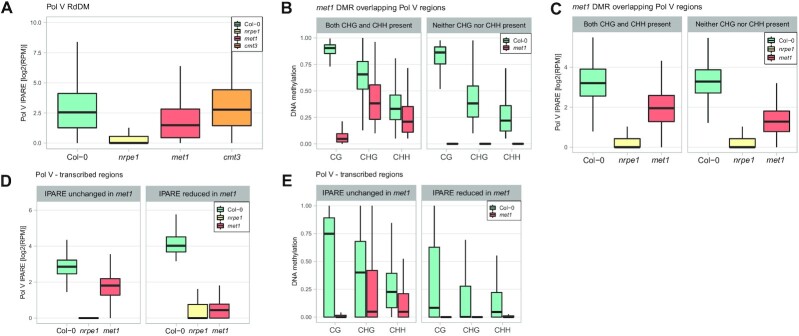
MET1 is needed for maintenance of RdDM at loci where it affects DNA methylation in all contexts. (**A**) Effects of DNA methyltransferase mutants on Pol V transcription. Pol V IPARE signal levels were plotted on previously identified Pol V RdDM regions (12) in Col-0, *nrpe1*, *met1* and *cmt3*. Individual biological replicates are shown in Supplementary Figure S4. (**B**) Control plot showing *met1* DMRs split by the presence or absence of non-CG methylation in *met1*. DNA methylation levels (42) were plotted on Pol V-transcribed *met1* CG DMRs split by the level of CHG and CHH methylation in *met1*. There were 2239 DMRs with both CHG and CHH present in both Col-0 and *met1* as well as 1035 DMRs with CHG and CHH present in Col-0 but absent in *met1*. DMRs were identified by difference between the WGBS CG signal of Col-0 and *met1* > 0.55 and FDR < 0.01. Presence of DNA methylation was defined as WGBS signal > 0.1 (CHG) or > 0.05 (CHH). Absence of DNA methylation was defined as WGBS signal of 0. (**C**) Substantial reduction of Pol V transcription in *met1* on loci that lose DNA methylation in all contexts in *met1*. Pol V IPARE signal was plotted on two categories of Pol V-transcribed *met1* DMRs in Col-0, *nrpe1*, and *met1*. (**D**) Control plot showing Pol V-transcribed genomic bins split by the presence or absence of MET1-dependent Pol V transcription. Pol V IPARE signal was plotted on regions with either Pol V IPARE reduced (2231 bins) or unchanged (5755 bins) in *met1*. Bins were identified as Pol V-transcribed by IPARE signal being significantly greater in Col-0 compared to *nrpe1* (FDR < 0.05 (36)). IPARE signal was defined as reduced in *met1* by *P* < 0.01 at 2-fold change or greater calculated using GFOLD (37), and as unchanged in *met1* by GFOLD *P* < 0.01 at 0.1-fold change or smaller and fold change smaller than 2. (**E**) Substantial reduction of DNA methylation in *met1* in all contexts on genomic bins with MET1-dependent Pol V transcription. DNA methylation levels (42) in CG, CHG and CHH contexts were plotted on regions with Pol V IPARE signal reduced or unchanged in *met1*.

The most prominent silencing pathway that overlaps RdDM is maintenance of CG methylation by MET1 (25). Disruption of this process by mutating *MET1* affects the levels of CHH methylation and has an impact on Pol V binding to chromatin (25). This pathway is likely to be responsible for high levels of CG methylation remaining in *nrpe1* and downstream mutants on RdDM Pol V-transcribed loci (Figure 1B). To test the impact of MET1 on Pol V transcription, we performed Pol V IPARE in the *met1* mutant. The overall accumulation of Pol V transcripts on all known RdDM Pol V-transcribed regions (12) was reduced in *met1* to a greater extent than in *drm2* or *cmt3* but was still strongly enriched over the background level observed in *nrpe1* (Figures 1A and 4A). This indicates that maintenance of CG methylation by MET1 is important but not absolutely required for Pol V transcription.

Our findings that loss of DNA methylation in all contexts in downstream RdDM mutants leads to reduction of Pol V transcription suggest a similar relationship in *met1*. To test this possibility, we found DMRs that lose CG methylation in *met1* (*met1* DMRs) and are transcribed by Pol V. We then split these DMRs into categories based on the presence or absence of CHG and CHH methylation in *met1* (Figure 4B) and calculated the abundance of Pol V transcription in those groups in Col-0 wild type and *met1* mutant (Figure 4C). Regions with no CHG and no CHH methylation in *met1* had a substantially greater reduction of Pol V transcription in *met1* than regions that retain CHG and CHH methylation in *met1* (Figure 4C). The level of Pol V transcription in *met1* on loci with no CHG and no CHH methylation in *met1* was significantly lower than on control loci (*P* < 10^–16^, Wilcoxon test). This indicates that loss of DNA methylation in all contexts in *met1* leads to a substantial reduction of Pol V transcription.

To further confirm the role of all DNA methylation contexts for maintaining high levels of Pol V transcription, we performed a reciprocal analysis. We identified Pol V-transcribed loci where Pol V transcription was unchanged in *met1* (Figure 4D). These loci lost CG methylation but retained substantial levels of CHG and CHH methylation in *met1* (Figure 4E). In contrast, loci with significantly reduced Pol V transcription in *met1* (Figure 4D) had strong reductions of DNA methylation in all sequence contexts, including CHG and CHH (Figure 4E). Levels of CHG and CHH methylation in *met1* at loci that lost Pol V transcription in *met1* were significantly lower than at loci where Pol V transcription was MET1-independent (*P* < 10^–199^ and *P* < 10^–291^ respectively, Wilcoxon test). This indicates that remaining CHG and CHH methylation allows maintaining Pol V transcription in *met1*. Reduction of Pol V transcription in *met1* at a subset of loci is associated with the loss of DNA methylation in all sequence contexts.

These results demonstrate that at a subset of loci, disruption of CG methylation maintenance in the *met1* mutant leads to loss of DNA methylation in all sequence contexts. This negatively affects the level of Pol V transcription and disrupts the maintenance of RdDM. This indicates that MET1 is involved in determining the level of Pol V transcription and therefore contributes to the maintenance of RdDM.

### CMT3 contributes to RdDM maintenance at a subset of loci

**Figure 5.**
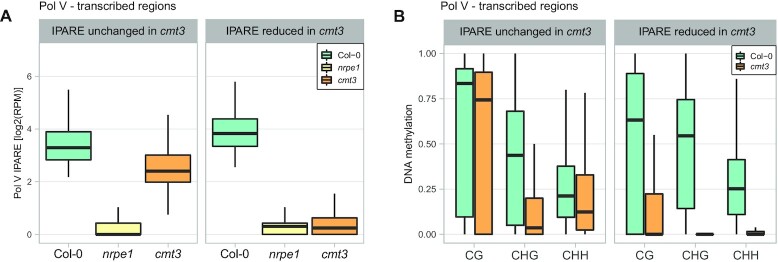
CMT3 affects RdDM maintenance at loci where it is needed for DNA methylation in all contexts. (**A**) Control plot showing Pol V-transcribed genomic bins split by the presence or absence of CMT3-dependent Pol V transcription. Pol V IPARE signal was plotted on regions with either Pol V IPARE reduced (912 bins) or unchanged (8735 bins) in *cmt3*. Bins were identified as Pol V-transcribed by IPARE signal being significantly greater in Col-0 compared to *nrpe1* (FDR < 0.05 (36)). IPARE signal was defined as reduced in *cmt3* by FDR < 0.05, and as unchanged in *cmt3* by FDR >0.9 and fold change smaller than 2. Individual biological replicates are shown in Supplementary Figure S5A. (**B**) Substantial reduction of DNA methylation in *cmt3* in all contexts on genomic bins with CMT3-dependent Pol V transcription. DNA methylation levels (42) in CG, CHG and CHH contexts were plotted on regions with Pol V IPARE signal reduced or unchanged in *cmt3*.

Although RdDM loci are also often targeted by CMT3 ((30) and Figure 1C), DNA methylated in CHG contexts is not preferentially bound by SUVH2 or SUVH9 *in vitro* (43). This predicts that CMT3 should not contribute to the maintenance of RdDM and mutating *CMT3* should not lead to the loss of RdDM Pol V transcription. To test this prediction, we identified Pol V-transcribed regions that had significant reductions of Pol V transcription in *cmt3* (Figure 5A, Supplementary Figure S5A). These sequences only partially overlapped loci with Pol V transcription dependent on AGO4, DRM2 or MET1 (Supplementary Figure S5B). We then compared them to regions with no change of Pol V transcription in *cmt3* (Figure 5A, Supplementary Figure S5A). Regions where Pol V transcription was unchanged in *cmt3* had a partial reduction of CHG methylation but retained high levels of CG and CHH methylation in *cmt3* (Figure 5B), higher than in *nrpe1* (Supplementary Figure S5C). In contrast, regions where Pol V transcription was significantly reduced in *cmt3* also had substantial reductions of DNA methylation in *cmt3* in all sequence contexts (Figure 5B), greater than in *nrpe1* (Supplementary Figure S5C). Levels of CG, CHG and CHH methylation in *cmt3* at loci that lost Pol V transcription in *cmt3* were significantly lower than at loci where Pol V transcription was CMT3-independent (*P* < 10^–142^ for CG, *P* < 10^–116^ for CHG, and *P* < 10^–234^ for CHH, Wilcoxon test). This indicates that CMT3 contributes to the maintenance of RdDM. At a subset of loci, disruption of CHG methylation maintenance in the *cmt3* mutant leads to loss of DNA methylation in all contexts, which disrupts the maintenance of RdDM.

### RdDM feedback is enriched on TE edges

**Figure 6.**
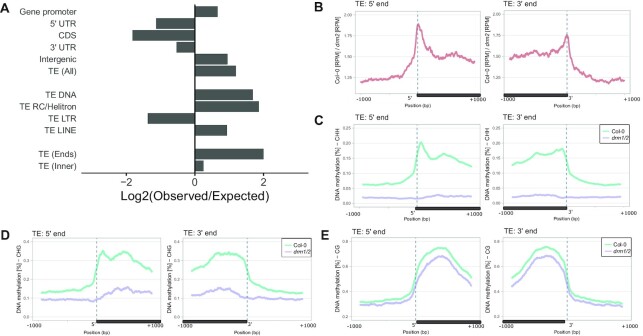
RdDM feedback is enriched on TE edges. (**A**) Overlaps of genomic bins that show evidence of DRM2-dependent Pol V transcription with genome annotations, including regions within genes, TE families, ends of long TEs and inner regions of long TEs (41). The plot shows ratios between observed overlaps and average expected overlaps calculated from 1000 permutations of random genomic bins. For all reported enrichments and depletions *P* < 0.001 (permutation test). (**B**) Average levels of DRM2-dependent Pol V transcription on 5′ and 3′ ends of TEs targeted by RdDM (41). (**C**–**E**) Average levels of DNA methylation (42) in the (C) CHH, (D) CHG and (E) CG contexts on 5′ and 3′ ends of TEs targeted by RdDM (41).

Edges of long TEs are known to be preferentially targeted by DRM2-dependent CHH methylation (30,42,44) and Pol V transcription, which has been proposed to act as a determinant of heterochromatin/euchromatin boundaries (10). In contrast, regions inside long TEs are primarily silenced by epigenetically maintained CHG and CG methylation (30,42,44). This suggests that edges of long TEs are likely to be targeted by stable silencing by the positive feedback of RdDM. To test this prediction, we identified genomic bins, where significant reduction of Pol V transcription in the *drm2* mutant indicates the presence of positive feedback by RdDM. We then overlapped these regions with genes and TEs. Distribution of loci with RdDM feedback resembled the overall pattern of Pol V transcription (10) in being enriched on intergenic regions and depleted on LTR TEs (Figure 6A). Importantly, it was more strongly enriched on edges of long TEs than on the inner regions of long TEs (Figure 6A). To further confirm that TE edges are preferential targets of the RdDM feedback, we plotted DRM2-dependent Pol V transcription on RdDM-targeted TEs (40,41). Average levels of DRM2-dependent Pol V transcription were enriched on edges of studied TEs (Figure 6B, Supplementary Figure S6AB), which is consistent with relatively low amounts of DNA methylation remaining on those regions in *drm2* (Figure 6C–E). This indicates that RdDM feedback is preferentially active on the edges of TEs, which is consistent with the role of RdDM in determining boundaries of heterochromatin.

## DISCUSSION

Our results indicate that RdDM is a self-reinforcing process, where Pol V transcription and DNA methylation enhance each other to maintain silencing. Presence of DNA methylation in at least one sequence context positively affects Pol V transcription and DNA methylation in multiple sequence contexts allows a crosstalk with other silencing mechanisms. Therefore, maintenance of DNA methylation on particular loci by MET1 and CMT3 pathways contributes to enhanced transcription by Pol V. Locus-specific contributions of individual silencing pathways are determined by a combination of the frequency of cytosines in particular contexts (31), presence of H3K9me2 (45) and other factors.

The mechanism of Pol V transcription enhancement by DNA methylation is unlikely to be mediated exclusively by Pol V recruitment as Pol V has been shown to transcribe broadly, even in euchromatin (12). Instead, DNA methylation may allow both Pol V recruitment and Pol V transcription at elevated rates, typical of RdDM loci (12). This is likely to be partially mediated by binding of methylated DNA by SUVH2 and SUVH9 and the recruitment of the DDR complex (25,26,43). However, these factors also contribute to the low level of non-RdDM Pol V transcription which indicates that the mechanism of Pol V transition from surveillance to RdDM transcription is likely to be more complex (12). More importantly, there are many loci in the genome which have high levels of DNA methylation but no evidence of RdDM Pol V transcription, such as genes with body DNA methylation (17). This indicates that DNA methylation is not sufficient to specifically control Pol V transcription. One potential explanation of the variable levels of Pol V transcription is exclusion of Pol V by Pol II and associated chromatin modifications. Another possibility is that there is an additional, yet unknown factor, which works together with DNA methylation to control the level of Pol V transcription.

Enhancement of Pol V transcription on methylated loci allows efficient recruitment of siRNA-AGO4 complexes to silenced loci (8–10) and facilitates further DNA methylation by DRM2 (17). Therefore, loss of AGO4 or SPT5L leads to the reduction of DNA methylation and consequent reduction of Pol V transcription. Enhancement of Pol V transcription on methylated loci is likely accompanied by recruitment of Pol IV and elevated production of siRNA, which explains why loss of downstream silencing factors leads to reduction of siRNA accumulation on subsets of loci (46,47).

Self-reinforcement of RdDM is particularly important on edges of TEs, which are preferentially transcribed by Pol V (10). This is consistent with the role of RdDM in precisely determining the boundaries between heterochromatin and euchromatin (10,48). The importance of RdDM self-reinforcement on TE edges may be explained by the low resolution of MET1 and CMT3 pathways, which is limited by the distribution of cytosines in symmetric contexts and/or the nucleosome size. In contrast, RdDM is enhanced by CHH methylation, which is more frequent and allows higher resolution of Pol V transcription determination (10). Pol V has also been shown to preferentially transcribe into TEs, which indicates that Pol V transcription may be enhanced by the proximity of euchromatin and heterochromatin, which could further contribute to precise determination of TE boundaries.

Our observations that MET1 and CMT3 are needed for elevated Pol V transcription at certain loci suggest that RdDM is efficiently maintained only if DNA methylation is above a certain threshold level. Loci where RdDM is capable of maintaining DNA methylation above this threshold may be silenced exclusively by RdDM. However, loci where RdDM cannot maintain DNA methylation above the threshold require at least one other silencing pathway for efficient silencing. The basis of this threshold mechanism remains unknown, however it is likely to integrate the level of Pol V transcription and the amount and properties of siRNA. This possibility is supported by the observation that tethering Pol V to the *FWA* locus leads to increased levels of DNA methylation (49). The mechanism of threshold is also likely to be controlled by a balance between DNA methylation and demethylation (50). The existence of such a threshold would be particularly important in *de novo* silencing as it would prevent inadvertent silencing of essential genes by low amounts of siRNA.

## DATA AVAILABILITY

High throughput sequencing datasets obtained in this study are available in Gene Expression Omnibus under accession number GSE168869.

## Supplementary Material

gkab746_Supplemental_File
