# Supplementary material for: Reinforcement of transcriptional silencing by a positive feedback between DNA methylation and non-coding transcription
Source: Nucleic Acids Res. 2021 Sep 1;49(17):9799–808. doi: 10.1093/nar/gkab746 (PMC8464056; doi:10.1093/nar/gkab746)
Supplement: gkab746_Supplemental_File [file gkab746_supplemental_file.pdf]

SUPPLEMENTAL FIGURE LEGENDS

Figure S1. Individual biological replicates of datasets showing small effects of mutants in downstream components of RdDM on Pol V transcription. Pol V IPARE signal levels were plotted on previously identified Pol V RdDM regions (1) in Col-0, *nrpe1* and *spt5l* (A) and Col-0, *ago4*, and *drm2* (B).

Figure S2. Maintenance of RdDM requires DNA methylation by DRM2 at loci not targeted by other silencing pathways.

A. Individual biological replicates of data showing a substantial reduction of Pol V IPARE signal in *nrpe1* on loci that lose DNA methylation in all contexts shown in Fig. 2B. Pol V IPARE signal was plotted on two categories of Pol V-transcribed *drm1/2* DMR in Col-0 and *nrpe1*.

B. Individual biological replicates of data showing a substantial reduction of Pol V IPARE in *drm2* on loci that lose DNA methylation in all contexts shown in Fig. 2B. Pol V IPARE signal was plotted on two categories of Pol V-transcribed *drm1/2* DMRs in Col-0 and *drm2*.

C. Individual biological replicates of control data showing genomic bins split by the presence or absence of DRM2-dependent Pol V transcription shown in Fig. 2C. Pol V IPARE signal was plotted on regions with either Pol V IPARE reduced or unchanged in *drm2*.

D. Substantial reduction of DNA methylation in *drm1/2* and *nrpe1* in all contexts on genomic bins with DRM2-dependent Pol V transcription. DNA methylation levels (2) in CG, CHG and CHH contexts as well as total DNA methylation levels were plotted on Pol V-transcribed regions with Pol V IPARE signal reduced or unchanged in *drm2*.

Figure S3. Downstream components are required for maintenance of RdDM at loci where they are needed for DNA methylation in all contexts.

A. Individual biological replicates of control data showing genomic bins split by the presence or absence of SPT5L-dependent Pol V transcription presented in Fig. 3A. Pol V IPARE signal was plotted on regions with either Pol V IPARE reduced or unchanged in *spt5l*.

B. Individual biological replicates of control data showing genomic bins split by the presence or absence of AGO4-dependent Pol V transcription presented in Fig. 3C. Pol V IPARE signal was plotted on regions with either Pol V IPARE reduced or unchanged in *ago4*.

Figure S4. Individual biological replicates of datasets showing minimal effects of mutants in DNA methyltransferase CMT3 on Pol V transcription shown in Fig. 4A. Pol V IPARE signal levels were plotted on previously identified Pol V RdDM regions (1) in Col-0 and *cmt3*.

Figure S5. CMT3 contributes to RdDM maintenance at loci where it is needed for DNA methylation in all contexts.

A. Individual biological replicates showing control data with genomic bins split by the presence or absence of CMT3-dependent Pol V transcription presented in Fig. 5A. Pol V IPARE signal was plotted on regions with either Pol V IPARE reduced or unchanged in *cmt3*.

B. Overlaps of genomic bins with Pol V transcription reduced in *nrpe1*, *met1*, *cmt3* and *ago4*.

C. Substantial reduction of DNA methylation in *cmt3* and *nrpe1* in all contexts on genomic bins with CMT3-dependent Pol V transcription. DNA methylation levels (2) in CG, CHG and CHH contexts as well as total DNA methylation levels were plotted on Pol V-transcribed regions with Pol V IPARE signal reduced or unchanged in *cmt3*.

Figure S6. RdDM feedback is enriched on TE edges.

A. Average levels of DRM2-dependent Pol V transcription on 5' and 3' ends of TEs targeted by RdDM (3) in biological replicate 1.

B. Average levels of DRM2-dependent Pol V transcription on 5' and 3' ends of TEs targeted by RdDM (3) in biological replicate 2.

SUPPLEMENTAL TABLE

Table S1. High throughput sequencing datasets obtained in this study. Experimental groups correspond to datasets generated in parallel from plants grown at the same time.

**Datasets**	**Exp. group**	**GEO acc.**	**Total reads**	**reads post-trimming**	**mapped reads**	**deduplicated reads**	**nuclear**
Col-0 IPARE	1	GSM4409524	Described previously in Tsuzuki *et al.* (2020)				
nrpe1 IPARE	1	GSM4409525	Described previously in Tsuzuki *et al.* (2020)				
spt5l IPARE	1	GSM4409526	Described previously in Tsuzuki *et al.* (2020)				
cmt3 IPARE	1	GSM5171710	15689618	5782750	3094943	2743344	2540409
Col-0 IPARE	2	GSM4409529	Described previously in Tsuzuki *et al.* (2020)				
ago4 IPARE	2	GSM4409530	Described previously in Tsuzuki *et al.* (2020)				
drm2 IPARE	2	GSM5171711	16778574	8329297	4508122	3475878	3114715
met1-3 IPARE	2	GSM5171712	15883353	7977408	4302897	2795337	2479618
Col-0 IPARE	3	GSM4409533	Described previously in Tsuzuki *et al.* (2020)				
nrpe1 IPARE	3	GSM4409534	Described previously in Tsuzuki *et al.* (2020)				

spt5l IPARE	3	GSM4409535	Described previously in Tsuzuki *et al*. (2020)				
ago4 IPARE	3	GSM4409536	Described previously in Tsuzuki *et al*. (2020)				
drm2 IPARE	3	GSM5171713	15851954	9500166	4382140	2946804	2514335
cmt3 IPARE	3	GSM5171714	16817100	9007012	3884136	2922802	2599268

SUPPLEMENTAL REFERENCES

1. Tsuzuki,M., Sethuraman,S., Coke,A.N., Rothi,M.H., Boyle,A.P. and Wierzbicki,A.T. (2020) Broad noncoding transcription suggests genome surveillance by RNA polymerase V. *Proc Natl Acad Sci U S A*, **117**, 30799–30804.

2. Stroud,H., Greenberg,M.V.C., Feng,S., Bernatavichute,Y.V. and Jacobsen,S.E. (2013) Comprehensive analysis of silencing mutants reveals complex regulation of the Arabidopsis methylome. *Cell*, **152**, 352–364.

3. Panda,K., Ji,L., Neumann,D.A., Daron,J., Schmitz,R.J. and Slotkin,R.K. (2016) Full-length autonomous transposable elements are preferentially targeted by expression-dependent forms of RNA-directed DNA methylation. *Genome Biology*, **17**, 170.

Figure S1

A

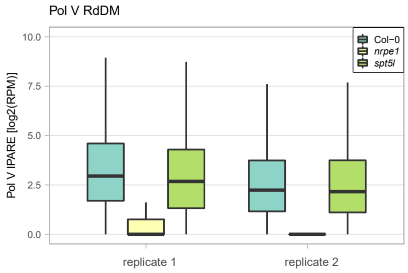
Pol V RdDM
Pol V IPARE [log2(RPM)]
10.0
7.5
5.0
2.5
0.0
Col−0
nrpe1
spt5l
replicate 1
replicate 2

B

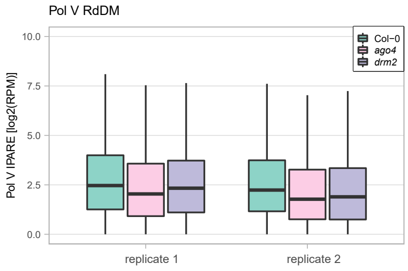
Pol V RdDM
Pol V IPARE [log2(RPM)]
10.0
7.5
5.0
2.5
0.0
Col−0
ago4
drm2
replicate 1
replicate 2

Figure S2

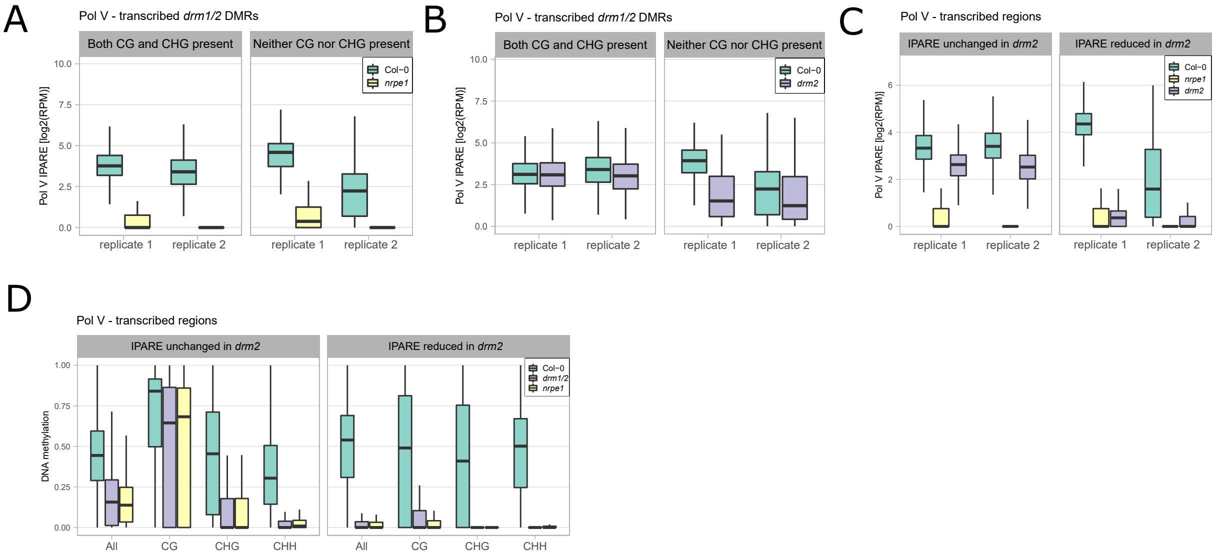
A
Pol V - transcribed drm1/2 DMRs
Both CG and CHG present
Neither CG nor CHG present
Col−0
nrpe1
Pol V IPARE [log2(RPM)]
replicate 1
replicate 2
B
Pol V - transcribed drm1/2 DMRs
Both CG and CHG present
Neither CG nor CHG present
Col−0
drm2
Pol V IPARE [log2(RPM)]
replicate 1
replicate 2
C
Pol V - transcribed regions
IPARE unchanged in drm2
IPARE reduced in drm2
Col−0
nrpe1
drm2
Pol V IPARE [log2(RPM)]
replicate 1
replicate 2
D
Pol V - transcribed regions
IPARE unchanged in drm2
IPARE reduced in drm2
Col−0
drm1/2
nrpe1
DNA methylation
All
CG
CHG
CHH

Figure S3

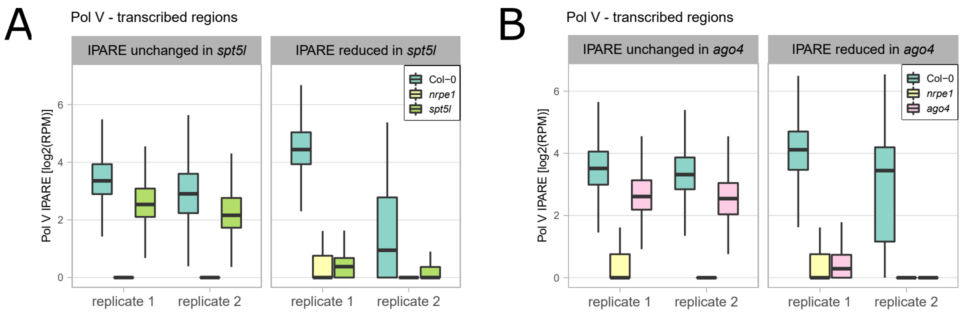
A
Pol V - transcribed regions
IPARE unchanged in spt5l
IPARE reduced in spt5l
Col−0
nrpe1
spt5l
Pol V IPARE [log2(RPM)]
0
2
4
6
replicate 1
replicate 2
replicate 1
replicate 2
B
Pol V - transcribed regions
IPARE unchanged in ago4
IPARE reduced in ago4
Col−0
nrpe1
ago4
Pol V IPARE [log2(RPM)]
0
2
4
6
replicate 1
replicate 2
replicate 1
replicate 2

Figure S4

A

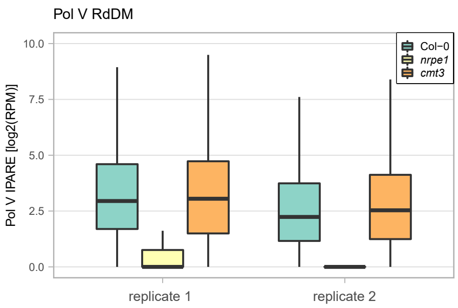

Pol V RdDM
Pol V IPARE [log2(RPM)]
10.0
7.5
5.0
2.5
0.0
Col−0
nrpe1
cmt3
replicate 1
replicate 2

Figure S5

A

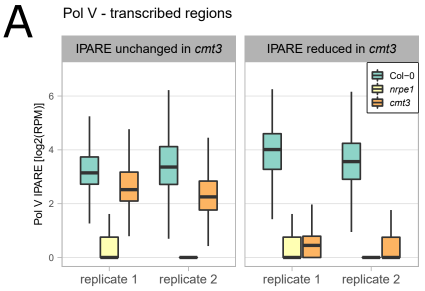

B

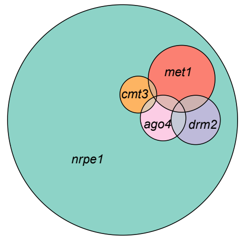

C

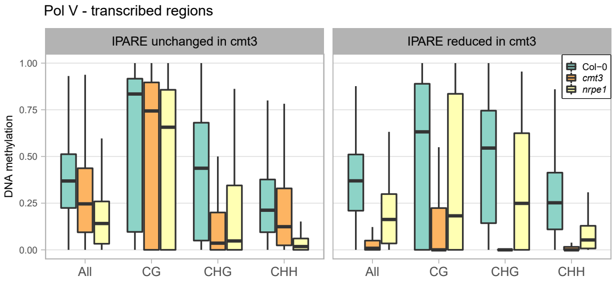

Figure S6

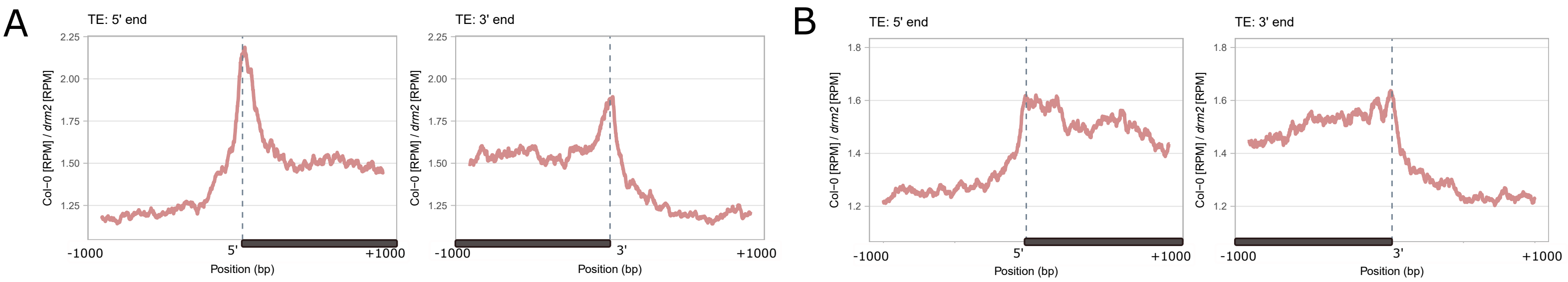
A
TE: 5' end
TE: 3' end
Col−0 [RPM] / drm2 [RPM]
2.25
2.00
1.75
1.50
1.25
-1000
5'
3'
+1000
Position (bp)
B
1.8
1.6
1.4
1.2
